# From sexless to sexy: Why it is time for human genetics to consider and report analyses of sex

**DOI:** 10.1186/s13293-017-0136-8

**Published:** 2017-05-03

**Authors:** Matthew S. Powers, Phillip H. Smith, Sherry A. McKee, Marissa A. Ehringer

**Affiliations:** 1 Institute for Behavioral Genetics, University of Colorado Boulder, Boulder, CO 80309 USA; 2 Department of Community Health and Social Medicine, CUNY School of Medicine, New York, NY 10031 USA; 3 Department of Psychiatry, Yale School of Medicine, New Haven, CT 06519 USA

## Abstract

Science has come a long way with regard to the consideration of sex differences in clinical and preclinical research, but one field remains behind the curve: human statistical genetics. The goal of this commentary is to raise awareness and discussion about how to best consider and evaluate possible sex effects in the context of large-scale human genetic studies. Over the course of this commentary, we reinforce the importance of interpreting genetic results in the context of biological sex, establish evidence that sex differences are not being considered in human statistical genetics, and discuss how best to conduct and report such analyses. Our recommendation is to run stratified analyses by sex no matter the sample size or the result and report the findings. Summary statistics from stratified analyses are helpful for meta-analyses, and patterns of sex-dependent associations may be hidden in a combined dataset. In the age of declining sequencing costs, large consortia efforts, and a number of useful control samples, it is now time for the field of human genetics to appropriately include sex in the design, analysis, and reporting of results.

## Background

In order to increase scientific rigor and reproducibility, Drs. Collins (Director, NIH) and Clayton (Director, Office of Research on Women’s Health) spearheaded an effort to require preclinical scientists to consider sex differences in their research [1]. A similar policy addressing the inclusion of women in clinical research has been in effect since 1993 [2]. Researchers are now working towards identifying how best to operationalize these policy initiatives across disciplines and disorders (e.g., [3–6]). The goal of this commentary is to raise awareness and discussion about how to best consider and evaluate possible sex effects in the context of large-scale human genetic studies. In particular, given the current rapid decline in whole genome sequencing costs, the relative affordability of high-throughput genotyping arrays, the establishment of large publicly available datasets and ongoing efforts of international consortia, the time is right to begin including sex as an important variable of interest.

Over the course of this commentary, we hope to accomplish four goals: (1) identify why sex differences are important to consider in the context of genetics; (2) establish evidence that sex differences are not being considered in human genetics; (3) provide compelling counter-arguments to the traditional reasons given for not considering sex differences in genome-wide association studies (GWAS); and (4) offer suggestions on how to test for sex differences in analyses and why it is important to include and report these analyses, no matter the result.

### Sex differences in genetics

Many fields in biology under-report sex differences and experiments that include this important variable are unfortunately low. Conducting and reporting sex differences in clinical and preclinical research have increased due to government policy, however, it remains an exception to the rule in science as a whole. One discipline, human genetics, has escaped the attention of government regulations and still largely ignores biological sex. There are a number of reasonable explanations for not including sex in human genetics studies (discussed later), but evidence across all levels of biomedical research show that ignoring sex also ignores an important biological context created by our genes, and within which our genes exist.

The most obvious difference between the sexes’ genomes is the presence of the Y-chromosome. Although most of Y-chromosome research to date primarily focuses on its role in sex determination and spermatogenesis (fertility), the advancement of molecular genetic tools has provided preliminary evidence that the Y-chromosome is involved in more than just sex determination and fertility. For example, the Y-chromosome has retained genes over evolution that are broadly expressed, dosage-sensitive, and encode proteins involved in chromatin modification, transcription, splicing, and translation. While a thorough discussion of the role of the Y-chromosome outside of the reproductive tract is beyond the scope of this commentary, we direct the reader to a review by Hughes and Page [7] that provides a compelling argument for why we should study the role of the Y-chromosome in the genetics of disease.

Along with the presence/absence of the Y-chromosome, males and females also differ in the number of X-chromosomes present. Until recently, very little information was generated about the X-chromosome’s relationship to complex human disease because most studies omitted it from analysis, or did the analysis incorrectly [8]. Past reasons for this omission include lack of coverage on genotyping chips, different number of genes on the X-chromosome versus the autosomes, and lack of statistical power to detect associations. With the advances in genotyping technology, though, microarrays now include thousands of markers on the X-chromosome compared to the arrays of old that include only a few. This increase in amount and quality of X-chromosomal data is beginning to uncover associations between X-linked genes and conditions like autoimmune disease [9], autism [10, 11], impaired cognitive function [12], and even alcoholism [13]. Further, genes on the X-chromosome are more likely to have sex-specific expression compared to genes on the autosomes [14, 15], which results from X-inactivation in females. However, the copy of the X-chromosome that is inactivated differs within each cell and some genes (~15%) on the second X-chromosome avoid X-inactivation altogether, which can lead to increased expression of the X-linked gene(s) in females compared to males [15]. Further, X-linked genes are all fully expressed in males, making them more likely than females to be influenced by X-linked genetic variations.

Chromosomal differences aside, males and females differ remarkably with regards to the structure of their genomes. For example, analysis of post-mortem human brains show that around 2.5% of all genes are differentially spliced and expressed between males and females [16], suggesting that the regulatory genome differs between sexes. Indeed, a number of studies across model species (e.g., flies, rodents) show mRNA expression level differences that depend on sex (e.g., [17–19]). To put this in perspective, unrelated human males share 99.9% of their genomes while unrelated males and females share only about 98.5% [16] (although this estimate is somewhat controversial). This could mean that the genome of a human male may be more similar to the genome of a male chimpanzee, sharing around 98.8% (e.g., [20, 21]) than between human males’ and females’ genomes. Despite these differences in the shared genome between males and females, sex differences in disease phenotype/genotype relationships are often not directly studied, even though there are many examples of diseases that differ in prevalence by sex.

Sex differences in gene expression patterns are important to highlight, as these differences can be due to genetic regulatory machinery like enhancers, binding sites for transcription factors, or sex steroid hormone receptors that predominantly exist in the non-coding (non-exonic) regions of the genome. Interestingly, these non-coding regions often include disease-associated single nucleotide polymorphisms (SNPs) (e.g., [22, 23]). Differences in the regulatory genome between sexes and the existence of disease-relevant SNPs in regulatory regions support the likely possibility that bi-directional relationships exist between genotype and phenotype that can depend on sex. To add further complexity, these bi-directional relationships can both exaggerate phenotypic variation between sexes, but also minimize differences, as is the case with genetic compensatory mechanisms (e.g., X-inactivation) [24]. Thus, if the statistical associations that capture such relationships are opposite in direction for female compared to male genomes, or substantially stronger for one sex compared to the other, a composite association may mask important relationships. Indeed, this has proven to be the case; a 2015 case/control candidate gene study of age-related macular degeneration (AMD) provided the first evidence of a disease association that is female-specific [25]. The analysis uncovered a SNP within the *DAPL1* gene, rs17810398, which is associated with AMD at the genome-wide significance level in females (*p* = 2.6 × 10^−8^), but not in males (*p* = 0.382). rs17810398 had not been previously identified as associated with AMD in large GWAS. While the functional implications of this sex-specific association between rs17810398 and AMD risk are still unknown, a more recent study by the same group identified another sex-specific locus related to copy number variation in the complement component 4A gene, which had a strong protective effect in females [26]. Results such as these highlight the cost of not performing sex-specific analyses—that information valuable to the health of both sexes may remain undiscovered.

The importance of considering sex in human genetic studies is not just limited to the realm of uncovering high-confidence associations between genotype and phenotype that would be hidden when sex is not considered. Ignoring potential sex-specific association both stalls scientific progress and can have even broader consequences. For example, we know that in vivo pharmacodynamics and pharmacokinetics can depend on sex (e.g., [27–29]), ethnicity (e.g., [30]), and genotype, but human geneticists often assess the impact of ethnicity and not sex. Important theoretical (e.g., population migration, natural selection, founder effect, etc.) and historical (e.g., subtle differences in ethnicity between case and control populations led to false positive findings) reasons underscore why geneticists assess the impact of ethnicity, but there are clear theoretical and evidence-based justifications for why assessing biological sex is important as well, as discussed above.

Another important reason for assessing and reporting analyses of sex relates to the impact that genetic association studies will have (and have had) on personalized medicine. As pharmacogenetics and other therapeutic approaches that are driven by individuals’ genotype become more prominent in healthcare, the consequences of not considering sex differences in treatment decisions has the potential to be harmful to not only the patient, but possibly the field of pharmacogenetics as a whole if public confidence in the field is diminished. To highlight potential consequences, we can consider an example of neuropathic pain. Emerging reports indicate that females with variants in the *MC1R* gene show analgesia to the kappa-opioid drug pentazocine, but males with the same variants do not [31]. Moreover, polymorphisms in the *OPRM1* gene are associated with pressure-related pain sensitivity in men but not in women [32]. Clearly, these sex-specific associations have implications for the efficacy of treatment options, but given the extreme dependence liability of opioid drugs, choosing a drug that will not be effective for its intended use in certain genotypes increases dependence liability while showing no treatment efficacy. Sex differences in drug efficacy alone should be enough to increase assessment and reporting of sex effects, but potentially increasing a negative outcome like drug dependence in one sex compared to the other is obviously an important consideration.

### Sex is not studied in human genetics

To gain insight into the proportion of human genetics studies that include sex differences in their analyses, literature searches were conducted using two search engines: PubMed and Web of Science (note that we did not curate or trim the search results in anyway). PubMed was used because it is the standard search engine in biomedical research, is human curated, and only searches titles/abstracts/keywords. Web of Science, like PubMed, is human curated and searches titles/abstracts/keywords, but has the added benefit of sorting results by the number of times an article is cited, allowing us to focus on only high-impact articles (defined as an article cited ≥100 times). Both searches were limited to human subjects.

The first literature search aimed to retrieve all papers that test for genetic associations, so the search terms were “genome-wide association study” OR (“genome-wide” AND “association”) OR “gwas” OR (“genome” AND “association”) OR (“exome” AND “association”) OR (“transcriptome” AND “association”) AND “humans” and returned a total of 32,779 results from PubMed, and 3039 from Web of Science (with ≥100 citations). The second search aimed to retrieve all genetic association publications that include mention of sex differences and/or analyses of sex, and therefore “sex difference” OR “by sex” OR “sex-specific” were added. This search returned a total of 168 results from PubMed (or 0.5% of all PubMed results) and 33 from Web of Science (or 1% of results from the most cited articles in the field). In addition to limiting our above search to “sex”-related terms, we repeated the search using the terms “X-chromosome” and “Y-chromosome.” Inclusion of the term “X-chromosome” yielded 195 articles from PubMed (0.6%) and 18 from Web of Science with ≥100 citations (0.6%). Inclusion of the term “Y-chromosome” yielded 63 articles from PubMed (0.19%) and 4 from Web of Science with ≥100 citations (0.13%).

These search results are surprising; 1% of genetic association study publications in either PubMed or Web of Science report anything related to sex differences in their searchable text and an even smaller proportion consider the sex chromosomes. The extremely low percentage of reported sex differences underscores the lack of attention to this subject in human genetics, and highlights the need for future studies to not only include sex as an important biological variable, but also report the result within the searchable text (i.e., title/abstract/keyword). To be fair, it is likely that sex differences are considered more often than are being reported in the literature. Unpublished results due to publication bias remain an unaddressed problem in science, and this ultimately contributes to wasted time and money. Not reporting sex differences, even if null, presents the same problem in that it restricts information that can be useful for other scientists.

### Why analyses of sex are often neglected in human genetic studies

Both clinical and preclinical researchers are routinely required to consider sex differences, but human genetic studies have largely considered sex only by regressing it out of statistical models. Most studies that assess sex in any way just include it as a covariate in their analyses, along with other relevant confounding variables (e.g., age). However, this approach does not directly assess whether genetic effects are dependent on sex, but instead provides a loose estimate of sex effects that may or may not be examined more closely. In other words, sex is often included as a control variable, and the variance attributable to sex is not closely examined; it is essentially parceled out and discarded.

Given the obvious importance of assessing sex in the context of experimental results, why is it that sex differences are not more frequently studied in human genetics? The likely answer is that a number of issues exist when conducting statistical analyses on genetic data, most of which are related to the size and complexity of the datasets generated, as well as the wide variation in selecting, defining, and measuring phenotypes. Because of this size and complexity, researchers often use simple statistical methods to uncover associations between genotype and phenotype. For example, traditional GWAS analyses typically examine >500,000 SNPs across the genome, leading to demanding multiple testing adjustment standards, setting the current standard for genome-wide significance at *p* < 5 × 10^−8^ [33]. Such a conservative *p* value threshold protects against detecting false positives; however, some researchers have suggested that the threshold is too conservative [34] and likely leads to high rates of false negatives, which can stall progress much like publication bias. The inherent issues of power and stringent *p* value thresholds has created a culture aimed at increasing power at all costs, and one of the casualties is little attention paid to sex.

The desire to maximize power also underlies the two reasons routinely cited to justify controlling for sex instead of directly assessing it. Not surprising, the main rationale is not enough statistical power to assess sex differences because the sample size has to be split in half. While it is often true that stratifying the data by sex will reduce statistical power, it is also true that power to detect an effect does not rely on sample size alone. Stratifying analyses by sex can result in increased power to detect significant genetic signals if the signals are small (or null) in one sex and larger in the other, or if large signals exist with opposite effects across sex [35]. In other words, if effect size is larger when split by sex than when sex is combined, statistical power may actually increase. Thus, the rationale of “not enough statistical power” becomes less justifiable.

The second reason used to justify controlling for sex is that genotyping/sequencing costs are prohibitive, making it extremely expensive to collect enough data to reach the sample size necessary to uncover sex differences. While this argument was once valid, the price of sequencing is rapidly declining, allowing for more samples to be collected and the all-important statistical power to increase. Further, large collaborative genetic consortia are collecting genotype/phenotype data and making it available to the scientific community at an increasingly rapid pace. As these datasets continue to expand, the availability of control/comparison samples as well as replication samples grows, again increasing statistical power. Declining sequencing cost combined with the growth of consortia and data sharing efforts should provide adequate sample sizes (at least control group samples) to start assessing sex in human genetics.

### Sex needs to be evaluated separately: best approach for evaluation of sex in genetic studies is to stratify

With the recent directive from NIH to consider sex as a variable of interest in preclinical studies, the best way to approach it in human genetic studies is not yet clear. Many investigators (and grant reviewers) still believe strongly that reducing sample size by dividing the sample is not a good option. We hope this commentary will serve as a starting point for these investigators to reconsider this stance, and that they will be involved in developing strategies to evaluate and report the impact of sex in their work. Until best practices for considering sex in human genetic studies are developed and refined, there are some steps that can be taken now.

One strategy is to simply be less stringent in the rules for power and multiple testing *p* value corrections, for exploratory purposes. Typically, a significant treatment × sex interaction is necessary to justify conducting a follow-up analysis stratified by sex. However, uncovering a significant interaction that passes correction for multiple testing in the realm of human genetics is difficult, unless there is a very large sex effect on an association between phenotype and genotype [36]. Thus, simply conducting the analyses allowing for some flexibility (less stringent with *p* values) often uncovers patterns in the data, and patterns in data can be just as informative as *p* values derived from association tests of single genetic loci. Reconsider the story of rs17810398 within the DAPL1 gene. Previous GWAS did not uncover an association between rs17810398 and age-related macular degeneration, but when analyses were stratified by sex, the association was highly significant for females (*p* = 2.6 × 10^−8^) and not males (*p* = 0.382) [25]. Significant associations can be lost when combining male and female data into one dataset.

Another strategy is to make use of publicly available datasets, which are expanding every day. However, this does not mean we need to wait for sample sizes to double before asking questions about sex differences. The Psychiatric Genomics Consortium (PGC) currently has a sample size of greater than 400,000 subjects with phenotypic information covering a wide range of psychiatric disorders [37]. Further, the PGC is committed to open sharing of data, and because the NIH requires datasets to be published along with manuscripts, recruiting enough individuals’ genotype information is no longer an insurmountable task, especially as data from “control” samples are growing rapidly (e.g., 1000 genomes—[38]; Precision Medicine Initiative®—[39]).

Even without access to the large publicly available databases, consistently conducting and reporting analysis on sex in all genetic association experiments can be beneficial to science in a number of ways. The first and most obvious benefit is the possibility of detecting a genome-wide significant result that depends on sex, even while lacking a large enough sample size to reach genome-wide significance in a combined male/female dataset. As mentioned above, stratifying analyses by sex can result in increased power to detect significant genetic signals if the signals are small (or null) in one sex and larger in the other, or if large signals exist with opposite effects across sex [35]. Further, a difference in variance (not means) between sexes is lost by a standard, fixed effect regression term for sex; therefore conducting analyses stratified by sex will improve power [40]. Finally, estimates of genetic associations with non-significant (genome-wide) *p* values can be useful to other researchers who use meta-analytical techniques to synthesize data across a large number of studies. By reporting the estimates of association stratified by sex, they become usable in meta-analyses, allowing for even more information to be gleaned over time.

Another strategy is for journal editors to require the reporting of sex statistics in order to publish, like many of the other specific requirements for reporting results. While helpful, requiring a separate section in all papers for reporting sex differences may be too extreme, but a few short sentences addressing the issue along with supplementary tables is an option. Indeed, there is a growing movement for journals to require the reporting of results by sex. In 2012 the Institute of Medicine published a report discussing the issue, and ultimately, the report recommends (among other things) that journals should require authors to present sex difference analyses when the study design allows [41]. A number of high impact journals (e.g., Lancet, Science, and Nature) have developed policies outlining the inclusion and reporting of sex differences (for a listing of journals and policies, see [42]), and it is only a matter of time before the major human genetics journals follow suit.

At this time, our recommendation is for all human genetics experiments to conduct stratified analyses on sex at all times, even without statistical justification in the form of a sex × SNP interaction. Simply conducting the analyses is not enough, though, as scientists will also need to report that the analyses were done and what they found, independent of the result. Furthermore, it is important for investigators who do observe evidence for sex differences to always include sex as a keyword in their manuscript, which would allow for a more comprehensive results list when searching the literature for sex differences.

## Conclusion

The goal of this commentary is to raise awareness and discussion about how to best consider and evaluate possible sex effects in the context of human genetic studies. Herein, we presented arguments for why sex differences are important to consider in the context of genetics, established evidence that sex differences are not being considered, and provided compelling counter-arguments to the traditional arguments for not considering sex differences in human genetics. Including sex in the design, analysis, and reporting of results will improve the transparency, rigor, and generalizability of genetic association studies, accelerating scientific progress. The inclusion of sex in genetic studies will ultimately improve our understanding of health and disease for both women and men.

## Abbreviation

GWAS: Genome-wide association studies
